# Imatinib increases oxygen delivery in extracellular matrix-rich but not in matrix-poor experimental carcinoma

**DOI:** 10.1186/s12967-017-1142-7

**Published:** 2017-02-23

**Authors:** Mikhail Burmakin, Tijs van Wieringen, P. Olof Olsson, Linda Stuhr, Aive Åhgren, Carl-Henrik Heldin, Rolf K. Reed, Kristofer Rubin, Carina Hellberg

**Affiliations:** 1 Ludwig Institute for Cancer Research, Science for Life Laboratory, Uppsala University, 751 24 Uppsala, Sweden; 2 Division of Vascular Biology, Department of Medical Biochemistry and Biophysics, Karolinska Institute, 171 77 Stockholm, Sweden; 3 School of Biosciences, University of Birmingham, Birmingham, B15 2TT UK; 4 Department of Laboratory Medicine, Medicon Village, Lund University, 223 63 Lund, Sweden; 5 Department of Biomedicine, University of Bergen, Bergen, Norway; 6 Centre for Cancer Biomarkers (CCBIO), University of Bergen, Bergen, Norway; 7 Department of Experimental Medical Science, Lund University, BMC D10, 22381 Lund, Sweden

**Keywords:** Hypoxia, Interstitial fluid pressure, Receptor tyrosine kinase, Tumor stroma

## Abstract

**Background:**

Imatinib causes increased turnover of stromal collagen, reduces collagen fibril diameter, enhances extracellular fluid turnover and lowers interstitial fluid pressure (IFP) in the human colonic carcinoma KAT-4/HT-29 (KAT-4) xenograft model.

**Methods:**

We compared the effects of imatinib on oxygen levels, vascular morphology and IFP in three experimental tumor models differing in their content of a collagenous extracellular matrix.

**Results:**

Neither the KAT4 and CT-26 colonic carcinoma models, nor B16BB melanoma expressed PDGF β-receptors in the malignant cells. KAT-4 tumors exhibited a well-developed ECM in contrast to the other two model systems. The collagen content was substantially higher in KAT-4 than in CT-26, while collagen was not detectable in B16BB tumors. The pO_2_ was on average 5.4, 13.9 and 19.3 mmHg in KAT-4, CT-26 and B16BB tumors, respectively. Treatment with imatinib resulted in similar pO_2_-levels in all three tumor models but only in KAT-4 tumors did the increase reach statistical significance. It is likely that after imatinib treatment the increase in pO_2_ in KAT-4 tumors is caused by increased blood flow due to reduced vascular resistance. This notion is supported by the significant reduction observed in IFP in KAT-4 tumors after imatinib treatment. Vessel area varied between 4.5 and 7% in the three tumor models and was not affected by imatinib treatment. Imatinib had no effect on the fraction of proliferating cells, whereas the fraction of apoptotic cells increased to a similar degree in all three tumor models.

**Conclusion:**

Our data suggest that the effects of imatinib on pO_2_-levels depend on a well-developed ECM and provide further support to the suggestion that imatinib acts by causing interstitial stroma cells to produce a less dense ECM, which would in turn allow for an increased blood flow. The potential of imatinib treatment to render solid tumors more accessible to conventional treatments would therefore depend on the degree of tumor desmoplasia.

## Background

Carcinomas characteristically have a dysfunctional stroma with a fibrotic extracellular matrix (ECM), aberrant blood vessels that leak plasma proteins, little or no lymphatic drainage and an elevated interstitial fluid pressure (IFP) [1]. The dense ECM forms a functional barrier for convective and diffusive transport, promotes malignant progression and has impaired blood flow [2–5]. The combination of rapidly proliferating neoplastic cells and a dysfunctional blood circulation results in tumor hypoxia that is associated with resistance to radiotherapy, as well as a limited efficacy of commonly used chemotherapeutic agents, contributing to poor patient prognosis [6–9].

The small tyrosine kinase inhibitor imatinib (Glivec, STI571) is licensed for treatment of chronic myeloid leukemia and gastrointestinal stromal tumors by virtue of the fact that it inhibits both the BCR-ABL kinase and the stem cell factor receptor kinase. It also inhibits the platelet-derived growth factor (PDGF) receptor tyrosine kinases, as well as the kinases of the colony-stimulating factor-1 receptor (CSF-1R) and the discoidin domain receptors [10, 11]. Long-term (days) treatment with imatinib lowers IFP in several carcinoma model systems [12–15].

The concentration and architecture of the collagen fibrous network, together with the hyaluronan/proteoglycan ground substance, determine the hydraulic conductivity in tissues [16]. In general, induced modulation of the density of collagen fibrils and fibril structure or network architecture, correlate with changes in IFP in experimental carcinoma [17–21]. Furthermore, the high concentrations of the glycosaminoglycan (GAG) hyaluronan and of collagen type I that are characteristic of pancreatic ductal carcinoma result in a high tissue pressure in these tumors [2]. A reduction in the levels of collagen and/or GAGs results in an improved blood flow leading to improved efficacy of chemotherapy [2, 4]. In the ECM-rich human colonic carcinoma KAT-4/HT-29 (KAT-4) xenograft model, imatinib lowers IFP, increases the interstitial fluid volume and the dynamic exchange between blood and tumor interstitium [13, 22, 23]. The GAG content in these tumors, however, was unaffected by imatinib treatment [23], underlining the notion that both GAGs and the collagen network in concert determine the hydrodynamic properties of tissues [16].

Imatinib reduces hypoxia in the A549 human lung adenocarcinoma, which over-expresses the PDGF β-receptor (PDGFR-β), as well as in the LS174T human colorectal adenocarcinoma xenograft mouse tumor models [14, 15]. In KAT-4 experimental carcinoma imatinib modulates the structure of the interstitial collagen network and increases collagen turnover in parallel to increasing the dynamic exchange between blood and the tumor interstitium, suggesting that imatinib alters tumor physiology through an effect on the tumor interstitial ECM [23]. In the present study where we used three different xenograft tumor models differing in amount of interstitial ECM, we describe the effects of imatinib on tumor IFP, tumor pO_2_, proliferation and apoptosis.

## Methods

### Cells

KAT-4 and CT-26 cells were from the American Type Culture Collection and maintained in RPMI 1640 medium containing 10% fetal bovine serum, 100 U/mL penicillin and 100 µg/mL streptomycin. The previously described B16F10 cells expressing PDGF-BB (B16BB) [24] were maintained in Dulbecco’s modified Eagle medium containing the supplements as above. KAT-4 cells were originally described as originating from a thyroid tumor [25], however, a thyroid origin of the KAT-4 carcinoma was later questioned and the cells were actually shown to be related to the human colorectal adenocarcinoma cell line HT-29 [26]. The origin of the KAT-4 cells was verified by Short Tandem Repeat loci analyses (IdentiCell, Aarhus, Denmark), and, as expected [26], KAT-4 matched with HT-29 although alleles D13S317:12 and TH01:9 were absent. The cell line has not undergone epithelial-to-mesenchymal transition [27]. Henceforth the KAT-4/HT-29 cells are referred to as KAT-4.

### Animals

KAT-4 (2 × 10^6^), CT-26 (10^6^), or B16BB (10^6^) cells in 100 μL PBS were injected s.c. into six to eight week-old Fox Chase SCID mice (M&B, Ry, Denmark). Treatment was commenced when KAT-4 tumors reached 400 mm^3^, and when CT-26 and B16BB tumors reached 200 mm^3^. Mice were randomized to receive *p.o.* vehicle (PBS) or 100 mg/kg/day imatinib for 4 days. All measurements were performed 4 h after the last drug administration. Animal experiments were approved by the local ethics committee at Uppsala University (Sweden) and Bergen University (Norway) and performed according to the UKCCCR guidelines [28].

### Measurement of IFP

IFP was measured using the ‘wick-in-needle’ technique, as previously described [29]. For each tumor, the IFP was determined by calculating the mean of three independent readings.

### Determination of extracellular and plasma volumes

Tumors and skin were analyzed based on the dilution principle with radiolabeled tracers, as described previously [18]. Tissue samples were dried at 50 °C for several weeks until no additional weight loss could be obtained, the final dry weight minus the initial wet weight was taken as total tissue water (TTW). After performing a bilateral functional nephrectomy, extracellular volume (ECV) was determined by measuring ^51^Cr-EDTA levels (Institute of Energy Technology, Kjeller, Norway), plasma volumes were determined via ^125^I-labeled human serum albumin (^125^I-HSA) Institute of Energy Technology, Kjeller, Norway). Distribution volumes were determined as plasma equivalent volumes from radioactivity in the tissue compared to radioactivity in plasma. ^51^Cr-EDTA was administered *i.v*. via the tail vein in a volume of 0.2 mL PBS (containing 300,000 cpm), 85 min before ^125^I-human serum albumin, in 0.2 mL PBS (3 × 10^6^ cpm), was injected using the same catheter. Animals were sacrificed 5 min later after heart puncture. Dorsal skin was sampled for reference. Radioactivity was measured using a COBRA II, Auto-gamma counter (Packard).

### Measurement of steady-state pO_2_ and blood flow

Tumor pO_2_ and blood flow were measured simultaneously utilizing two-channel probes using OxyLite 2000 and OxyFlo instruments, respectively (Oxford Optronix, Oxford, UK). Four probes were inserted at different sites in each tumor and steady-state pO_2_ was recorded for 10 min.

### Hydroxyproline determination

The content of hydroxyproline in excised tumors was determined calorimetrically. Briefly, whole tumors were minced using scissors and hydrolyzed in 6 M HCl at 120 °C for 4 h and the resulting hydrolysates were analyzed for hydroxyproline content essentially as described earlier [30].

### Antibodies

Goat anti-mouse CD31 antibody (used at 4 µg/mL) was obtained from Santa Cruz Biotechnology (Santa Cruz, CA). Monoclonal antibodies against Ki-67 (TEC3; 1:50), α-smooth muscle cell actin (ASMA) (clone 1A4; 0.7 µg/mL), and desmin (1:50) were from DAKO (Glostrup, Denmark). An antibody against MHC class II (1:200) and rabbit antiserum against cleaved caspase-3 (1:200) were from Cell Signaling Technology (Danvers, MA). The rabbit polyclonal NG2 (1:250) antibody was obtained from Chemicon (Temecula, CA). The rabbit immunoglobulin G fraction against PDGFRβ [31] was used at 4 µg/mL. Biotinylated antibodies against mouse, rabbit and goat immunoglobulins (1:500) were from DAKO.

### Immunohistochemistry and stereological analyses of tumor blood vessels

Sections were de-paraffinized and pre-treated by boiling in 10 mM citrate buffer, pH 6.0, or in high pH target retrieval solution (DAKO). After quenching in 3% H_2_O_2_, slides were blocked in 20% serum species-matched to the secondary antibody. Staining was developed using DAB (Vector Laboratories, Burlingame, CA) or NBT/BCIP (Roche, Basel, Switzerland). Collagen fibers were visualized using 0.1% Sirius Red. Stereological quantification of capillary tumor blood vessels was performed after CD31 and ASMA, PDGFRβ, NG2 or desmin staining, using an eyepiece grid for unbiased counting, as described earlier [32]. Stereological quantification of CD31-positive vessels was performed using Leica QWin Standard digital image software and values for 10–40 fields of vision (0.09 mm^2^) were averaged. The fraction of cleaved caspase-3 positive cells or Ki67 positive cells was determined after analyzing 1000 cells from all tumors in each group.

### Statistical analyses

Statistical analysis of the IFP measurements and tumor growth assays was performed with one-way analysis of variance, with a subsequent post hoc analysis with Duncan’s adjustment. Results from TTW, extracellular volume and plasma volume measurements were analyzed using the Student’s t-test. p < 0.05 was considered as being statistically significant. Data are presented as mean ± 1 SD unless otherwise specified.

## Results

### Stromal cells and collagen

**Fig. 1:**
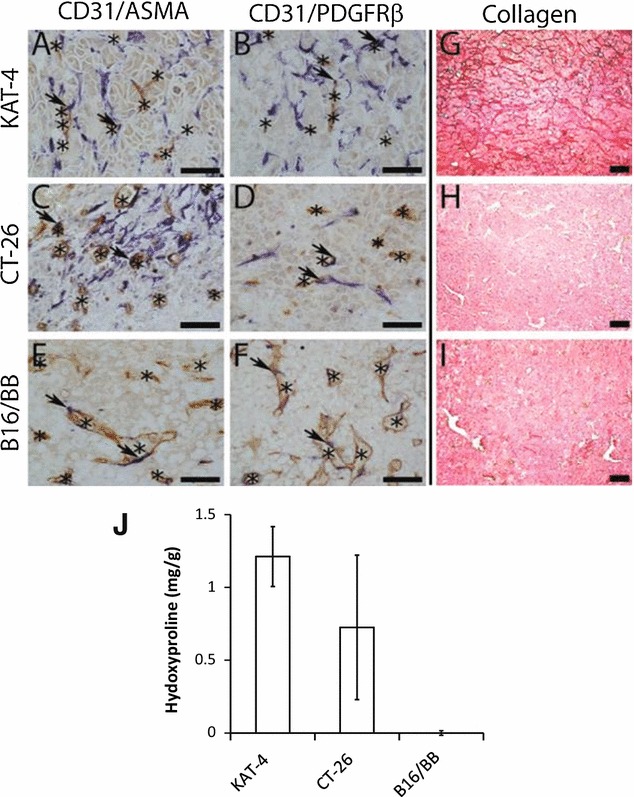
Stroma characteristics of KAT-4, CT-26 and B16BB tumors. KAT-4, CT-26 and B16/BB tumor sections were stained with antibodies directed against CD31 (**A**–**F** *brown*), ASMA (**A**, **C**, **E** *blue*) or PDGFRβ (**B**, **D**, **F** *blue*). CD31-positive structures are indicated *asterisk* and perivascular staining of ASMA and PDGFRβ is indicated by *black arrows*. Collagen fibers were stained by Sirius red (**G**–**I**). The bars represent 100 μm. Average hydroxyproline levels in whole tumors (**J**)

In the present study we employed tumor models where PDGFRβ is expressed in the stroma but not by the tumor cells, i.e. KAT-4, CT-26 and B16BB tumors (Fig. 1). B16BB cells exogenously expressing PDGF-BB were used since imatinib does not affect the growth rate of tumors generated from this clone [24]. These tumor models recruit stroma of different composition when grown in vivo (summarized in Table 1). Thus, the stroma of CT-26 and KAT-4 tumors contained ASMA- and PDGFRβ-positive fibroblasts (Fig. 1A–D), whereas B16BB tumors contained no apparent fibroblast stroma (Fig. 1E, F). KAT-4 deposited a well-developed collagen matrix in contrast to B16BB and CT-26 tumors (Fig. 1H, I). All three tumors were well vascularized, but KAT-4 tumor tissue had a higher density of CD31-positive structures than did CT-26 and B16BB (Fig. 1A–F). The recruitment of α-smooth muscle actin (ASMA)- and PDGFRβ-positive pericytes was similar in the three tumor types (Fig. 1A–F). Hydroxyproline levels in hydrolysates from whole tumors, a measure of total tumor collagen content, were 1.21 ± 0.21 (n = 4) and 0.73 ± 0.50 (n = 4, ±SD) mg/g wet weight carcinoma tissue in KAT-4 and CT26 carcinomas, respectively, whereas hydroxyproline was not detected in B16BB melanoma (Fig. 1J). In previous reports we have characterized the collagen fibril network in KAT-4 and CT-26 carcinomas and reported average fibril diameters of around 45 nm in both tumor types [20, 23].

**Table 1:** Semi- quantitative analysis of the stroma components in KAT-4, CT-26 and B16BB tumors

Parameter	KAT-4	CT-26	B16BB
Abundance of blood vessels	+++	++	++
Abundance of perivascular ASMA	+	+	+++
Abundance of stromal ASMA	+++	+++	−
Abundance of perivascular PDGFR-β	+	+	+++
Abundance of stromal PDGFR-β	+++	+	+
Collagen	+++	++	−

### Interstitial fluid pressure

**Fig. 2:**
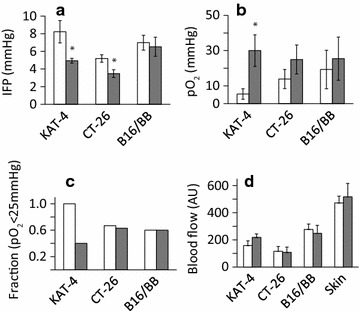
Imatinib reduces IFP in KAT-4 and CT-26 carcinomas. Mice bearing subcutaneous tumors were treated for 4 days with vehicle (n = 11; *white bars*) or 100 mg/kg imatinib (n = 8; *grey bars*). All measurements were made 4 h after the last administration. **a** Tumor IFP was measured using the wick-in-needle technique. Data are averages from at least 7 different tumors in each group. **b** Oxygen pressure was averaged for the individual tumor (at least three measurements) and the data shown is the average of these averages. KAT-4, n = 5 treated and 5 untreated, CT-26, n = 9 treated and 8 untreated, B16BB n = 5 treated and 5 untreated. **c**. The fraction of oxygen pressure with pO_2_ ≤ 25 mmHg measured in the same tumors as in B. **d** Blood flow was averaged for each tumor and the data presented is the average of the averages calculated for each tumor (in turn being the average of at least three successful measurements). KAT-4, n = 5 treated and 5 untreated, CT-26, n = 9 treated and 8 untreated, B16BB n = 5 treated and 5 untreated. Statistical analysis: **a**, **b** one-way ANOVA with Duncan adjustment; **c** Mann–Whitney U Rank; **d** Chi Square with Yates correction for continuity. Statistically significant differences (p < 0.05) compared to vehicle treatment are indicated *asterisk*

It is well established that treatment with 100 mg/kg imatinib for 4 days reduces IFP in a variety of carcinomas [12–14, 22]. This is in agreement with our present data showing that imatinib significantly reduced IFP in KAT-4 carcinomas from on average 8 mmHg in controls to 4.5 mmHg (≈44% decrease) after treatment (Fig. 2a). Similarly, imatinib lowered IFP in CT-26 carcinomas from an average of 5–3.5 mmHg (≈30% decrease), whereas IFP in B16BB tumors was not affected by treatment with imatinib (Fig. 2a).

### Tumor oxygen pressure (pO_2_)

**Fig. 3:**
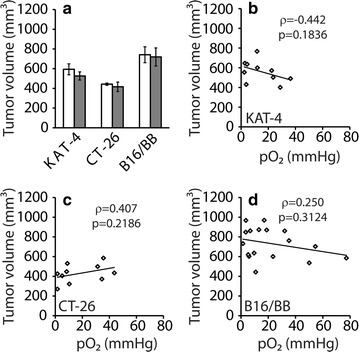
Tumor volume and pO_2_ do not correlate with one another. **a** Mean tumor volume ± SEM of tumors from mice treated for 4 days with vehicle (*white bars*) or 100 mg/kg imatinib (*grey bars*). **a** Mean tumor volume ± SEM of tumors from mice treated for 4 days with vehicle (*white bars*) or 100 mg/kg STI571 (*grey bars*). The numbers of analyzed tumors in vehicle and imatinib treatment groups, respectively, were KAT-4 (5 and 5), CT-26 (9 and 8) and B16BB (5 and 5). Tumor volume and pO_2_ were plotted for individual KAT-4 (**b**), CT-26 (**c**) and B16BB (**d**) tumors. The p-values for Spearman rank-order correlation coefficients (ρ), are given

The pO_2_ was on average around 5 mmHg in KAT-4 carcinomas prior to imatinib treatment and increased to about 30 mmHg with imatinib (Fig. 2b) (p < 0.05). For CT-26 a similar pattern was seen with an increase from pO_2_ of 15 mmHg in control to 25 mmHg occurring after imatinib treatment, although these changes in pO_2_ levels were not significant (Fig. 2b). Furthermore, all recorded pO_2_ values in vehicle-treated KAT-4 were below 25 mmHg in control carcinomas while after treatment with imatinib the fraction of pO_2_ values recorded below 25 mmHg was reduced by 60% (Fig. 2c). In control CT26 carcinomas and B16BB melanomas the fraction of pO_2_ below 25 mmHg was 0.6, and this was seen not to be lowered by imatinib treatment. Red blood cell velocities measured by laser Doppler flowmetry, taken as an indirect measure of local blood flow, were measured in the same tumors as those investigated in Fig. 2a–c. Red blood cell velocities were not significantly affected in any of the tumor models following treatment with imatinib (Fig. 2d). There was, however, a trend towards an increased blood flow (at least measured by the technique indicated here) after imatinib treatment of KAT-4 carcinomas (Fig. 2d). We did not find any significant correlation between tumor volumes and pO_2_ in any of the three model systems investigated (Fig. 3).

### Interstitial volume, local blood flow, total tissue water and plasma volume

Previously we have shown that treatment with imatinib increases ECV in KAT-4 carcinomas [22]. By contrast, in CT-26 carcinomas imatinib treatment resulted in a lowering of ECV from 1.22 ± 0.32 (n = 4) in control to 0.71 ± 0.22 mL/g dry weight (n = 3, p = 0.051) (Table 2). Total tissue water averaged 5.14 ± 0.58 (n = 4) in control and 4.76 ± 0.16 (n = 3) mL/g dry weight after imatinib treatment (p = 0.033). Plasma volume averaged 0.12 ± 0.05 (n = 4) in control and 0.09 ± 0.03 (n = 3) mL/g dry weight after imatinib (p = 0.40). The absolute changes in TTW corresponded to the changes in ECV suggesting that the intracellular volumes were not changed. Local blood volume in KAT-4 carcinomas, measured as the 5 min distribution volume for ^125^I-albumin, was not affected by treatment with imatinib [22].

**Table 2:** Extracellular volume (ECV), plasma volume (PV) and total tissue water (TTW) measured in CT-26 carcinomas treated with PBS (control) or imatinib

Treatment	TTW (mL/g dry weight)	PV (mL/g dry weight)	ECV (mL/g dry weight)
Vehicle	5.14 ± 0.58	0.12 ± 0.048	1.22 ± 0.29
Imatinib	4.76 ± 0.17	0.086 ± 0.035	0.71 ± 0.22

### Vessel characteristics

**Fig. 4:**
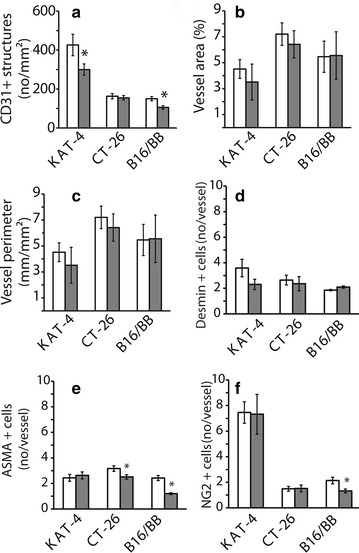
Analyses of vascular characteristics. Vascular characteristics were investigated in tumor sections prepared from mice treated for 4 days with vehicle (*white bars*) or 100 mg/kg imatinib (*grey bars*). For quantification we used at least 10 random fields of sections per tumor. **a** The number of CD31 positive blood vessels, **b** perimeter of CD31-positive blood vessels, **c** mean area of CD31-positive blood vessels area per vessels, **d** desmin-positive cells (number per CD31-positive vessel), **e** ASMA positive cells (number per CD31-positive vessel) and **f** NG2 positive cells (number per CD31-positive vessel). Data are presented as mean ± SEM and statistically significant differences (Student’s t-test; p < 0.05) compared to vehicle treatment are indicated by *asterisk*. The numbers of tumors in vehicle and imatinib treatment groups, respectively, were: KAT-4 (12 and 13), CT-26 (7 and 6) and B16/BB (7 and 7)

To investigate the possibility that imatinib reduces hypoxia by altering vessel function, we carefully explored if any vascular changes correlated with the ability of imatinib to increase pO_2_ in KAT-4 carcinomas. Imatinib reduced the number of CD31-positive structures in KAT-4 and B16BB tumors but had no effects in CT-26 tumors (Fig. 4a). Transcapillary fluid transport is affected by the area of the exchange vessels and the permeability for diffusive and convective transport. One relevant histologic parameter is the area of exchange, i.e. the area taken up by blood vessels relative to the total area (Fig. 4b). The relative vessel area was lower in KAT-4 carcinomas than in CT-26 and B16BB. Importantly, the relative vessel areas were not significantly affected by treatment with imatinib in any of the tumor models. Furthermore, we found no effect of imatinib on either vessel area or perimeter (Fig. 3b, c) [22]. Imatinib had no effect on blood vessel coverage of desmin-positive cells (Fig. 4d), whereas it significantly reduced the number of ASMA-positive cells per vessel structure in CT-26 and B16BB tumors (Fig. 4e), and NG2-positive cells in B16BB tumors. Imatinib had no observable effect on coverage of desmin-, ASMA- or NG2-positive cells in KAT-4 tumors (Fig. 4d–f).

### Cellular effects

**Fig. 5:**
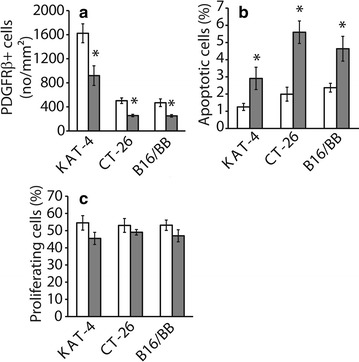
Imatinib decreases the number of PDGFRβ positive cells and increases tumor cell apoptosis. Tumor sections from mice treated for 4 days with vehicle (*white bars*, n = 7) or 100 mg/kg imatinib (*grey bars* n = 7) were stained with antibodies against PDGFRβ (**a**), as well as cleaved caspase-3 (**b**) and Ki-67 (**c**) to monitor apoptosis and proliferation. Data are presented as mean ± SEM. Statistically significant differences (Student’s t-test; p < 0.05) compared to vehicle treatment are indicated *asterisk*

We observed that imatinib, as expected, decreased the number of PDGFRβ-positive cells by approximately 50% in all three tumor types (Fig. 5a). Since imatinib inhibits the PDGFRβ kinase these data show that imatinib indeed was active in all three tumor models. Furthermore, imatinib significantly increased the number of apoptotic cells as judged by cleaved caspase-3 staining (Fig. 5b), while cell proliferation (Fig. 5c) was unaffected.

## Discussion

In this study, where we compared three murine malignant tumor models, we show that pO_2_ levels in tumor tissues at steady-state correlate with the amount of collagen in the stroma. Thus, stroma-rich KAT-4 carcinomas had six times lower pO_2_ levels than B16BB melanomas where no collagenous stroma could be detected. Treatment with imatinib, which affects collagen architecture in KAT-4 carcinoma [23], significantly increased pO_2_ in KAT-4, but had no significant effect on pO_2_ in the stroma-poor CT-26 carcinomas, or in B16BB melanomas. Similarly, imatinib had no effect on IFP in B16BB melanomas, but significantly reduced IFP in the other two tumor model systems, moreso in KAT-4 than in CT26 carcinomas.

Tumor interstitial pO_2_ is the result of oxygen provided to the tissue minus that used in cellular metabolism [33]. Our approach measured the pO_2_ in the tumor tissue directly, i.e. supply minus metabolism. Reports on the effect of imatinib on metabolism and oxygen consumption are scarce. Edema and dyspnea are the major adverse effects of prolonged imatinib treatment in patients. Such effects have been attributed to cardiotoxicity causing an adverse influence on heart mitochondria [34, 35], although this view has in fact been disputed [36, 37]. An uncoupling, or reduction of mitochondrial activity would reduce oxygen consumption and potentially increase pO_2_. The plasma C_*max*_ of imatinib in male ICR mice was determined to be 17 μM 2 h after administration of 100 mg/kg *p.o.* [38]. The plasma half-life of imatinib in mice was determined to be 2.3 h [38], whereas in humans it is around 10 h [39]. Will et al. [37] showed that imatinib, in doses below the expected plasma C_*max*_ in mice, had little or no effect on ATP production by cultured rat heart H9c2 undifferentiated myoblasts, neither when grown in glucose-containing medium nor in galactose-containing medium, which causes a shift in metabolism to oxidative phosphorylation. These authors also reported that respiration in isolated rat heart mitochondria was not affected by imatinib when the drug was administered in clinically relevant doses [37]. Furthermore, imatinib inhibits the expression of the glucose transporter Glut1 in BCR-ABL-positive, but not in BCR-ABL-negative, chronic myeloid leukemia cells [40]. Imatinib decreases glucose uptake from the media by suppressing glycolytic activity and increasing mitochondrial Krebs cycle activity in cultured cells [40]. Such an effect by itself should in fact cause a lowering of pO_2_, which was in contrast to that observed in the present study. Our present data show that cellular proliferation was unaffected in all three tumor models investigated by a 4-day treatment period with imatinib and, in addition, that pO_2_ levels tended to be similar in all three models after imatinib treatment. This then suggests that the increased pO_2_ levels in KAT-4 and CT-26 carcinomas were not due to changes in cell metabolism with decreased oxygen consumption. The fractions of apoptotic cells increased after imatinib treatment in all three tumor models but remained below 6% also after treatment. Imatinib induces or sensitizes several types of cells for endoplasmic reticulum stress and to the effect of reactive oxygen species [35–37], potentially explaining the observed increase in the fractions of apoptotic cells. Thus, the findings presented herein strongly suggest that imatinib, in the dose used, does not affect oxygen consumption but rather increases delivery of oxygen to the tumor tissue.

The amount of oxygen provided to a tissue is determined by the blood flow. Oxygen delivery to the tissue is flow-limited, while transport from blood to tissue occurs by diffusion and therefore is not a limiting factor in determining tissue pO_2_. The present data on vascular gross morphology in KAT-4 carcinomas and the effects of imatinib are in line with previously published data [22, 23], showing that vascular parameters are only marginally affected by imatinib treatment and cannot explain the significantly increased delivery of oxygen in these tumors. In the CT-26 carcinomas the pO_2_ levels also increased after treatment with imatinib although this increase did not reach statistical significance. The parameters of vascular morphology investigated in these carcinomas were unaffected by treatment with imatinib. Blood flow measured by laser Doppler is based on measurement of red cell velocity and thus does not give a true indication of blood flow in mL/min/g tissue. Although the method has a great advantage of being non-invasive, it is challenging in the current experimental approach when one has to compare the same tissue several days apart, and under conditions where vessel architecture is changing (cf Fig. 4a–f). Nevertheless, we recorded a trend towards an increased blood flow in KAT-4 carcinomas upon imatinib treatment, although the differences did not reach significance due to a large inter- and intra-tumor variation. Taken together, our data strongly suggest that the increased pO_2_ levels recorded after treatment of especially KAT-4 carcinomas is due to an increased blood flow. An increased blood flow must in turn mean that the resistance to flow is reduced. The reduced IFP in KAT-4 and CT-26 tumors after imatinib treatment would be expected to cause reduced inflow hindrance to the tumor and thereby result in an increased delivery of oxygen to the tissues.

## Conclusion

We have previously reported that imatinib alters the structure of the collagenous ECM in KAT-4 tumors and at the same time lowers IFP and increases the dynamic exchange of solutes between the tumor interstitium and the blood [12, 23]. The present study confirms that in tumor models that have a well-developed extracellular matrix, imatinib induces a lowering of interstitial pressure, and it further demonstrates that it improves tumor oxygenation by causing a recovery of blood flow. Further, the results reported here allow us to conclude that imatinib treatment has a potential value in rendering solid tumors of high desmoplasia to becoming more accessible to conventional treatments.

## Abbreviations

ASMA: α-smooth muscle actin
ATP: adenosine tri-phosphate
ECM: extracellular matrix
ECV: extracellular volume
DAB: 3,3′-diaminobenzidine
IFP: interstitial fluid pressure
GAG: glycosaminoglycans
PBS: phosphate buffered saline
PDGF: platelet-derived growth factor
SD: standard deviation
TTW: total tissue water
